# An interactive holographic projection system that uses a hand-drawn interface with a consumer CPU

**DOI:** 10.1038/s41598-020-78902-1

**Published:** 2021-01-08

**Authors:** Takashi Nishitsuji, Takashi Kakue, David Blinder, Tomoyoshi Shimobaba, Tomoyoshi Ito

**Affiliations:** 1 Faculty of Systems Design, Tokyo Metropolitan University, 6-6 Asahigaoka, Hino, Tokyo, 191-0065 Japan; 2 Garduate School of Engineering, Chiba University, 1-33 Yayoicho, Inage-ku, Chiba 263-8522 Japan; 3 Department of Electronics and Informatics (ETRO), Vrije Universiteit Brussel (VUB), Pleinlaan 2, 1050 Brussel, Belgium; 4 IMEC, Kapeldreef 75, 3001 Leuven, Belgium

**Keywords:** Displays, Computer science

## Abstract

Holography is a promising technology for photo-realistic three-dimensional (3D) displays because of its ability to replay the light reflected from an object using a spatial light modulator (SLM). However, the enormous computational requirements for calculating computer-generated holograms (CGHs)—which are displayed on an SLM as a diffraction pattern—are a significant problem for practical uses (e.g., for interactive 3D displays for remote navigation systems). Here, we demonstrate an interactive 3D display system using electro-holography that can operate with a consumer’s CPU. The proposed system integrates an efficient and fast CGH computation algorithm for line-drawn 3D objects with inter-frame differencing, so that the trajectory of a line-drawn object that is handwritten on a drawing tablet can be played back interactively using only the CPU. In this system, we used an SLM with 1,920 $\times$ 1,080 pixels and a pixel pitch of 8 μm × 8 μm, a drawing tablet as an interface, and an Intel Core i9–9900K 3.60 GHz CPU. Numerical and optical experiments using a dataset of handwritten inputs show that the proposed system is capable of reproducing handwritten 3D images in real time with sufficient interactivity and image quality.

## Introduction

Electro-holography is a very promising technology for three-dimensional (3D) display systems, because it is possible to reproduce fully the light reflected from an object using a spatial light modulator (SLM). However, the enormous calculation requirements for producing computer-generated holograms (CGHs), which are displayed on an SLM to modulate the incident light, are a significant problem for practical applications (e.g., for an interactive 3D display for a car navigation system). Consequently, there have been many studies of fast CGH calculation methods, such as look-up table (LUT)-based^1–5^, sparsity-based^6–8^, polygon-based^9–12^, and hardware-based^13–15^ approaches. With the evolution of both hardware and algorithms, the required computational complexity has been greatly reduced, and computational speed has been improved dramatically compared to the early days of this area of research. However, to achieve practical computing speeds, high-performance computers–such as graphics processing units (GPUs) or field-programmable gate arrays (FPGAs)–are still required.

On the other hand, many research topics aimed at improving the performance of holographic displays-e.g., enlarging the viewing angle^16,17^, projecting full-color images^18,19^, or implementing interactions on the display system^20,21^-are active domains of research, because holographic displays are expected to be effective visual interfaces. For example, Sando et al. proposed a 3D holographic-display system with a digital micromirror device (DMD) and rotating mirrors. They calculated CGHs using a 3D fast Fourier transform (FFT) based algorithm and succeeded in projecting 360$^\circ$ viewable 3D video at 10 Hz using a GPU with mouse and keyboard interactions^20^. Yamada et al., proposed an interactive, full-color holographic video system with finger-gesture input^21^. They captured finger gestures with leap motions and used an input interface for rotate-and-zoom operation. Although such interactive holographic-display systems have been successfully implemented at the laboratory level, they usually require GPU-based computation; therefore, miniaturization and power saving remain significant challenges.

**Figure 1:**
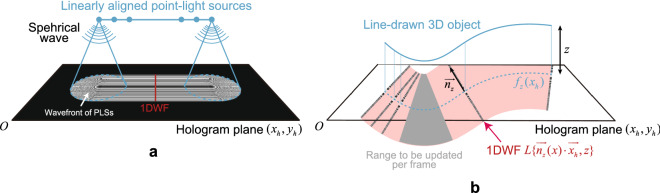
Overview of the CG-line method. (**a**) Principle of obtaining 1DWF. (**b**) Method to create CGH using the CG-line method in the proposed system.

Recently, the present authors have reported a fast CGH calculation algorithm for projecting a 3D object comprised of line-drawn objects^22^. Figure 1 shows the overview of the algorithm. Our algorithm, called the “Computer Graphics (CG)-line method” in this paper, utilizes the wavefront from linearly aligned point-light sources (PLSs) at the same depth to converge into a wavefront that is compressible in one-dimension (1D). We call this pattern as 1D wavefront (1DWF) in this paper. That is, instead of superimposing two-dimensional PLS wavefronts, as in the conventional LUT method, we have succeeded in greatly improving the computational speed by superimposing 1DWFs along the normal direction of a line. However, we did not previously create an interactive system using this method, and the computation speed was not fast enough to generate smooth interactive playback.

This paper proposes a holographic 3D display system that interactively displays the outlines of handwritten characters and basic shapes in the air. In this system, we applied inter-frame subtraction to the CG-line method to improve the computational speed and produce an interactive display working on a CPU. For animation that gradually completes a 3D image, such as handwritten input, the CGH is obtained by integrating the results of the holographic calculations for the input between frames. Therefore, by applying inter-frame subtraction to the 3D video, the amount of computation performed at each frame can be limited, and the computation time can be reduced significantly. The proposed system can be applied to 3D display systems with augmented-reality that overlay simple information or instructions on the human’s field of view (e.g., a car navigation system or a remote operational support system for workers) with attention-getting animations (e.g., strokes or splits).

**Figure 2:**
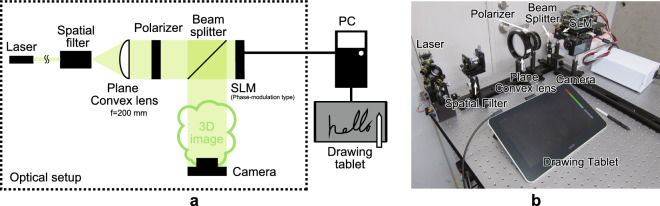
Optical setup of the proposed system. (**a**) Schematic illustration of the proposed system. (**b**) Optical setup of the proposed system.

## Results

Figure 2(a) shows a schematic illustration of the proposed system, and Fig. 2(b) shows an image of the laboratory setup for this system. We used a phase-modulation-type SLM (Holoeye AG, ‘HEO 1080P’) with a 1,920 $\times$ 1,080 pixel resolution and a pixel pitch of 8 μm $\times$ 8 μm. The modulation quantization has 256 levels (8 bits), and the refresh rate is 60 Hz. A green-light laser (Light Vision, JPM-1-3-(A4)APC) with a wavelength of 532 nm is used as the light source to project a monochromatic image, and a drawing tablet (Wacom One, DTC133) is the interactive interface employed to input progressively varying, hand-written, line-drawn 3D objects. The 3D image is projected in front of the SLM as a real image to enhance its visibility, and it can be observed on the image sensor of the camera. Note that the 3D image can be converted easily into a virtual image by changing the sign in the equation for the CGH calculation. We used the following computer environment: Microsoft Windows 10 Enterprise operating system; Intel Core i9–9900K 3.60 GHz CPU and 64 GB DDR4–2666 memory; Microsoft Visual C++ 2017 compiler with single floating-point computational precision.

**Figure 3:**
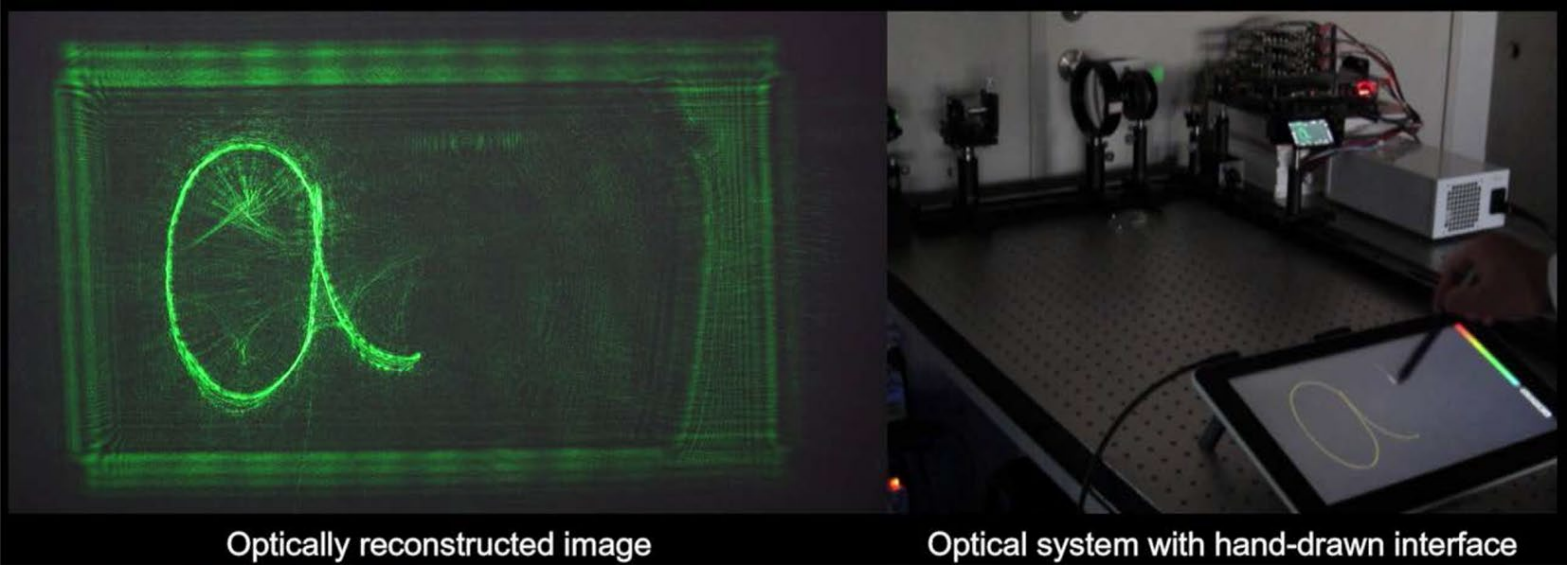
Example of drawing and projecting a 3D image.

**Figure 4:**
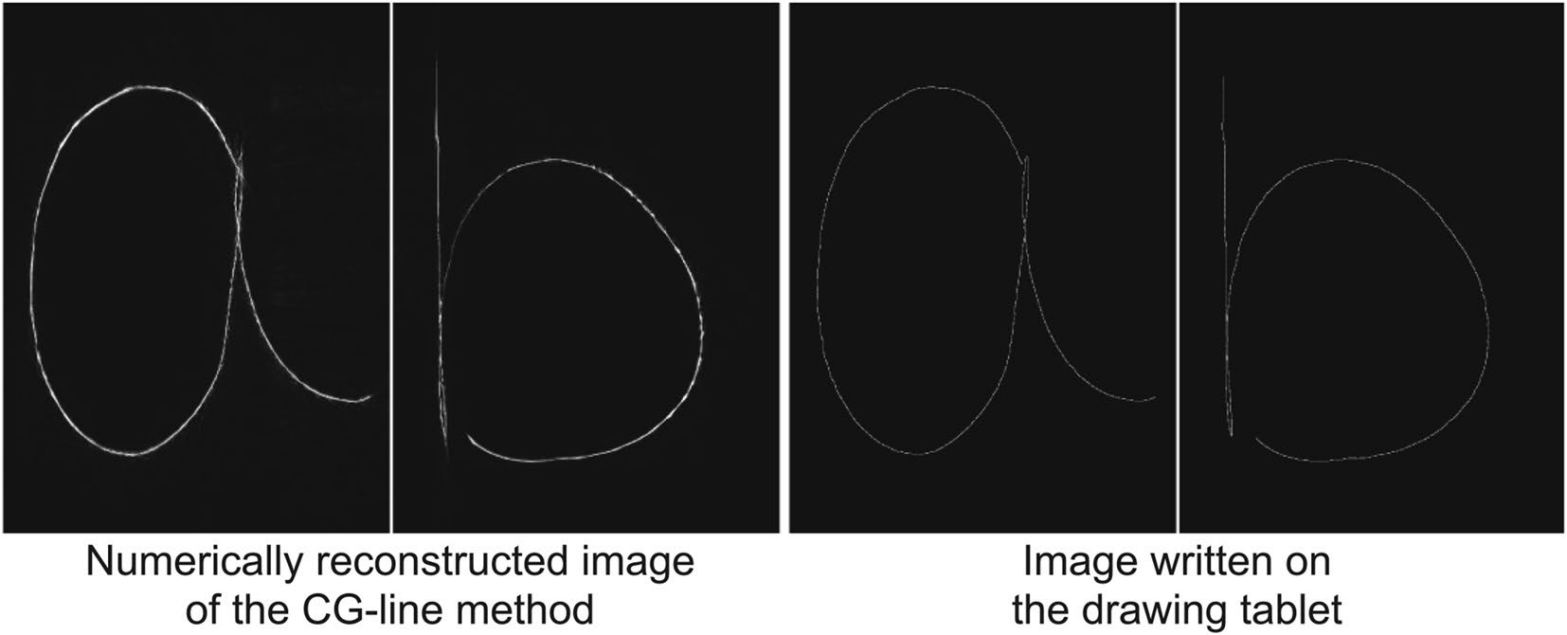
Numerical simulation of the reconstructed image of the hand-written letters ’a’ and ’b’.

Figure 3 and the Supplementaly video 1 show an example of an interactive 3D projection (The hand-written letters ‘a’ and ‘b’,were 0.25 m and 0.16 m, respectively, in front of the SLM in real time). The frame-rate of the 3D picture was set as variable but achieved 60 fps. As the supplemental video shows, there is no significant delay or lag between input and display. To check the image quality, we compared the peak signal-to-noise ratio (PSNR) and the structural similarity (SSIM) of numerically reconstructed images from the CGHs obtained using the proposed method with the images written on the drawing tablet , which we considered as ground truth. The result of the numerical simulation is shown in Fig. 4. The PSNR of ‘a’ is 25.8 dB while that of ‘b’ is 28.8 dB, and the SSIM of ‘a’ is 0.696 while that of ‘b’ is 0.748. In general, it is difficult to distinguish between two images with a PSNR greater than 30 dB, so the 3D image quality of this system is almost sufficient .

In order to generalize the results above, we conducted a comparative experiment on the response performance and image quality of 3D projections of handwriting input. We used a public dataset of hand-written input data^23^ with three CGH computation algorithms (the N-LUT method^1^ with frame subtraction, an FFT-based angular-spectrum method (ASM)^24^, and the CG-line method^22^ with frame subtraction). Here, we did not apply any iterative algorithm for optimizing the generated wavefront (e.g., Gerchberg-Saxton algorithm^25^ ) in every CGH computation algorithms because such algorithms are time consuming and thus not desirable for our intended application. The dataset was downloaded using a 13.3-in. tablet PC, and it contains the timestamps of control-point coordinates for 20 subjects drawing 16 gestures 10 times at three different speeds (slow, normal, and fast). We selected datasets for 10 subjects (Nos. 10, 11, 12, 22, 28, 68, 71, 88, 95, and 98) at fast speed for the first try; i.e., we prepared 160 datasets in total. Since the original dataset was written in too small a region, for this experiment the coordinates and the input time were multiplied by a factor of 5; i.e., we enlarged the input coordinates while keeping the drawing speed.

**Figure 5:**
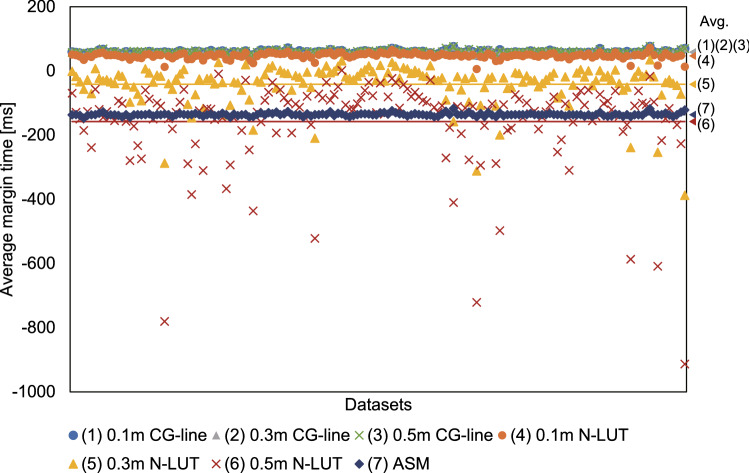
Results of the margin-time tests for different CGH calculations with each of the datasets.

First, we measured the margin of CGH computation time between sequential handwritten inputs in the datasets; if the CGH computation time is longer than the input interval, the 3D display does not properly follow the hand-written input. Here, the update period of the CGH from the complex-amplitude distribution was set to the interval of the timestamp contained in the dataset. This corresponds to the sampling rate of the drawing tablet, which we argue to be a reasonable setting. Figure 5 shows the average margin time for each calculation method and for datasets with different reconstruction depths (0.1 m, 0.3 m, and 0.5 m), and Table 1 shows the average margin time for all datasets. Here, a positive margin time means that the CGH calculation time is shorter than the input interval. Further, since the ASM computation time depends only on the resolution, the average margin time was calculated from the ASM execution time for an aperture image of 1,920 $\times$ 1,080 pixels.

In applying the CG-line method with frame subtraction, almost all of the margin times were positive and sufficiently large, except for a few extreme data that can be attributed to noise. On the other hand, when the N-LUT method was applied, the margin time was positive when the reconstruction distance was 0.1 m; however, for the cases at 0.3 m and above the margin time became larger and more negative. This is because the computational cost of the N-LUT method is positively correlated with the reproduction distance. The average margin times were also negative in the ASM case.

**Table 1:** Average margin times for all datasets.

[ms]	CG-line	N-LUT	ASM
0.1 m	58.5	46.7	
0.3 m	58.5	$-40.5$	$-136$
0.5 m	58.6	$-155$	

**Figure 6:**
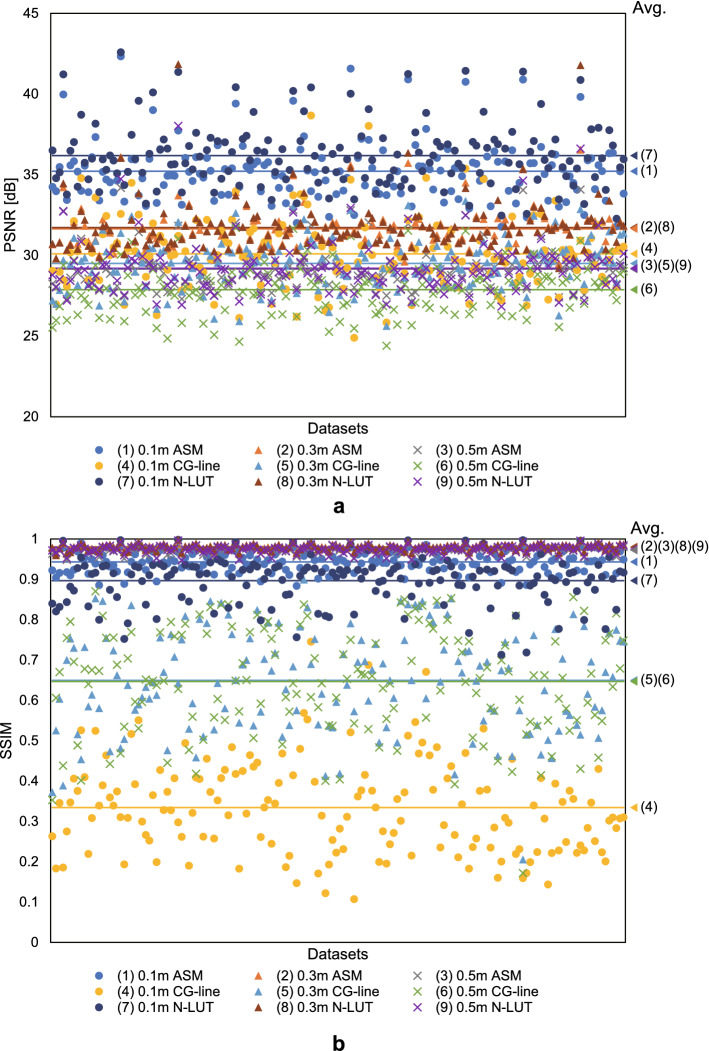
Results of image-quality evaluation with the different datasets. (**a**) PSNR. (**b**) SSIM.

**Figure 7:**
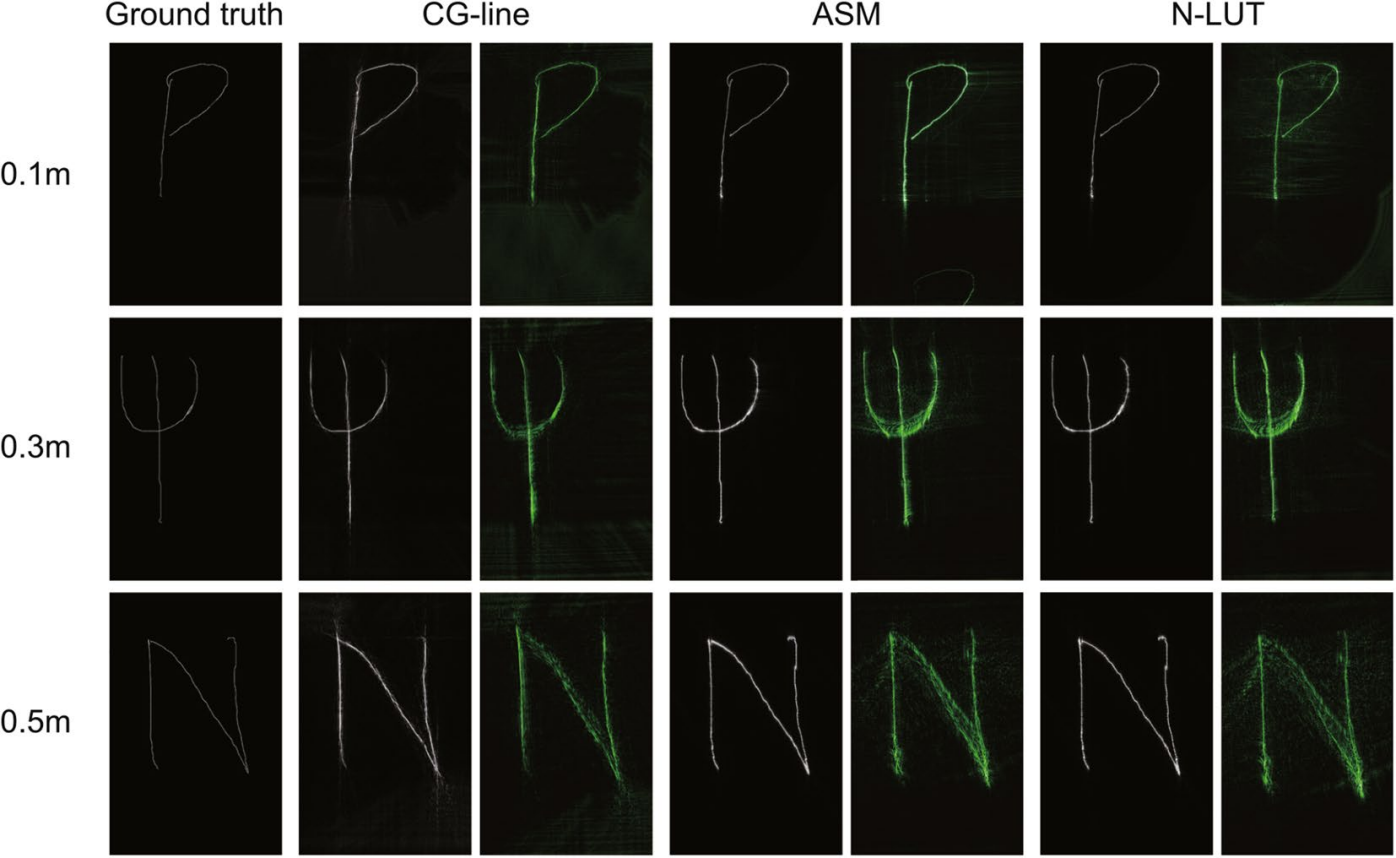
Examples of the reconstructed images. Left columns: numerical simulations. Right columns: optical experiments.

Second, we measured the average PSNR and SSIM of each image numerically reconstructed from the CGHs created with the CG-line method , with N-LUT method and with ASM, using the images written on the drawing tablet as a ground truth. Note that the CGHs were used after completing the input and drawing for each dataset. Figure 6 shows the PSNR and SSIM for each reconstruction distance and dataset, and Table 2 shows the average PSNR and SSIM for all datasets. Although the PSNR and SSIM of the CG-line method are inferior to those of the ASM, at each depth the PSNR of the CG-line method is close to or exceeds 30 dB, which is the standard for high-quality 2D images. Figure 7 shows a reproduction simulation and an example of an optically reconstructed image. Here, we observed that the reproduction distance of the optically reproduced image was displaced by about 0.2 m for the 0.5 m reproduced image and 0.08m for the 0.3 m reproduced image for all the calculation methods, due to the problem in the optical system such as unintentional diffraction in the half-mirror. However, numerical simulations have shown that the image formation is correct, and this issue can be corrected in the future by precise adjustment of the optical system.

**Table 2:** Average PSNR and SSIM for all datasets.

	PSNR [dB]			SSIM		
	CG-line	N-LUT	ASM	CG-line	N-LUT	ASM
0.1 m	30.1	36.2	35.2	0.334	0.897	0.943
0.3 m	29.5	31.7	31.6	0.650	0.980	0.980
0.5 m	27.9	29.2	29.2	0.647	0.975	0.972

**Figure 8:**
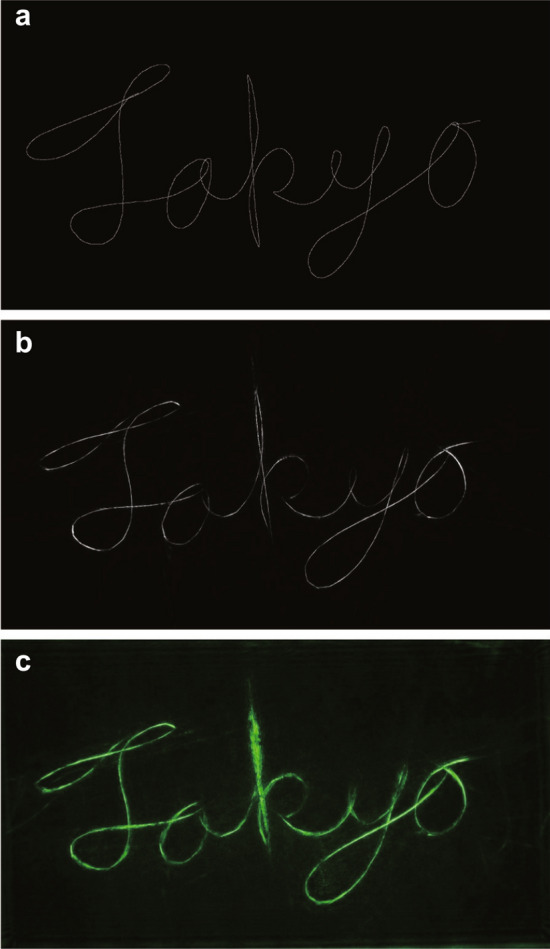
Reconstructed images of “Tokyo”. (**a**) Image written on the drawing tablet. (**b**) Numerical simulations. (**c**) Optical experiments.

In addition to the above experiments, we also conducted more practical experiments in which English and Japanese words are projected at the same depth with the proposed method. Supplementary video 2 shows the results of the optical experiments, and Fig. 8 shows a part of the results. In this experiment, “Tokyo”, “Tokyo” in Kanji, “HELLO” and “Hello” in Japanese Hiragana were drawn at 0.16m from the SLM, and they were projected in real-time with sufficient quality to be recognized. The results of the image quality evaluation by numerical simulation are shown in Table 3.

**Table 3:** PSNR and SSIM in experiments of drawing English and Japansese word.

	PSNR [dB]	SSIM
“Tokyo”	25.6	0.814
“Tokyo” in Kanji	27.1	0.841
“HELLO”	27.2	0.885
“HELLO” in Japanese hiragana	27.5	0.873

## Discussion

In terms of computation time, a sufficient processing-time margin is obtained by applying the CG-line method and frame subtraction. We calculated this using a dataset obtained from an experiment conducted with multiple subjects using a generic drawing tablet; therefore, we can say definitively that the proposed system can display 3D images with sufficient interactivity at normal writing speed.

**Figure 9:**
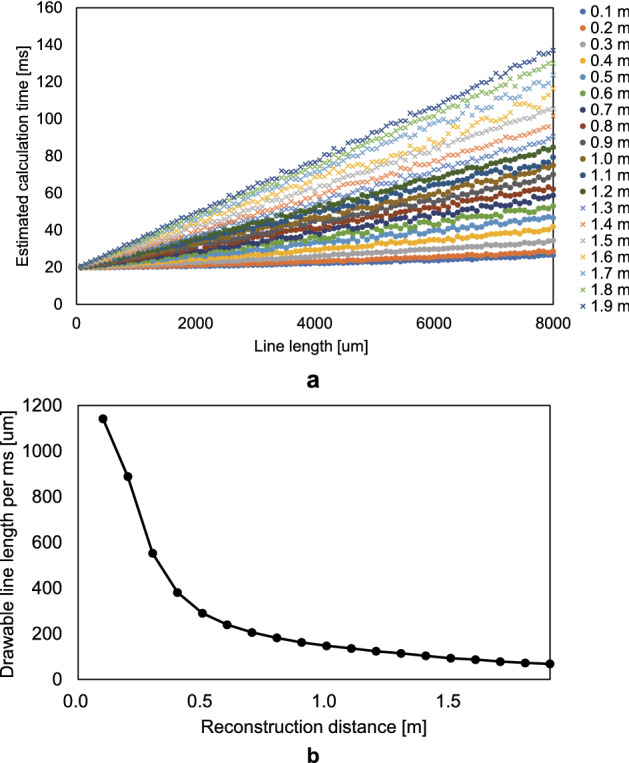
Computational performance of the CG-line method. (**a**) Relation between CGH calculation time and line length. (**b**) Drawable line length per millisecond as a function of the reconstruction distance.

In order to clarify the computational performance of the proposed system, we also investigated the relationship between line length and CGH computation time. The results are shown in Fig. 9. In this experiment, the computation of complex-amplitude distributions using the CG-line method and its conversion to CGH were evaluated separately in order to understand the influence of the line length on the computation speed. For the computation of the complex-amplitude distribution, we want to prevent incorrect evaluation of the computational speed in cases for which the 1DWF data lies (partially) outside of the CGH region and is thereby ignored. To address this issue, we prepared a complex-amplitude buffer that can accommodate the whole domain of the 1DWF, and we computed complex-amplitude distributions for straight-line-drawn objects with lengths ranging from 8 μm to 8 mm (1 to 1000 pixels on a drawing tablet) and for depths of 0.1 m to 1.9 m. For the conversion to CGH, we calculated the average processing time for converting complex-amplitude distributions for 1920$\times$1080 pixels to CGHs, which was 19.4 ms.

As shown in the Fig. 9(a), the processing time is approximately proportional to the line length, and the farther away the playback distance is, the longer is the required processing time. This is due to the increasing length of the 1DWF considering aliasing on the SLM as the playback distance increases. The average input length per millisecond of dataset used in this study was 0.438 pixels (3.504 μm on the SLM), suggesting that the proposed system is capable of reproducing 3D images with sufficient interactivity at 1.9 m or less, as shown in Fig. 9(b).

Table 1 shows that the computation time of the CG-line method has less influence on the increase in the playback distance than that of the N-LUT method. As shown in Fig. 9(a), the computation time of the CG-line method is positively correlated with the playback distance as well as the N-LUT method. However, since the CG-line method synthesizes 1D wavefronts, whereas the N-LUT method synthesizes 2D wavefronts, the effect of the increase in computation time on the increase in distance is small. Therefore, under the conditions of this paper, the margin time of the CG-line method is almost invariant to the playback distance.

In the case of FFT-based CGH generation methods such as ASM, the computation time depends on the resolution, as shown in Table 1. On the other hand, the computation time of the proposed method depends almost entirely on the total line length of the object. Therefore, the FFT-based method is more advantageous for projecting complex objects, while the proposed method is suitable for simple objects. For practicality and versatility, we should adopt a method that adaptively switches the computation method depending on the complexity of the object in the future. We are currently developing an algorithm for switching the computational methods.

In terms of image quality, the proposed method is inferior to the ASM and N-LUT methods. However, it shows a PSNR that is close to the high-image-quality standard. In addition, from the simulation and optical reproduction experiments, it is clear that the proposed system achieves sufficient image quality, considering that the 3D images can be recognized clearly.

## Conclusion

We have proposed an interactive 3D projection system based on electro-holography that runs on a consumer CPU with a drawing tablet. By limiting the projectable 3D images to multiple 2D images composed of outlines and applying inter-frame subtraction to the CG-line method, we have constructed a system with sufficient interactivity even with ordinary CPUs. Since our system’s computational cost depends mainly on the complexity of the 3D image and not on the resolution of the CGH, it is suitable for the projection of 3D images that add depth to simple figures such as symbols and letters. Therefore, our system is expected to be applied to car navigation systems and remote work support systems.

On the other hand, it is still difficult to draw 3D objects such as polygon meshes because the current algorithm cannot draw continuous lines in the depth direction. Besides, since approximation is added to the CGH creation process, the image quality is degraded compared to the conventional method. Therefore, the improvement of the proposed method’s expressiveness and image quality with high speed is critical issues to be addressed in the future.

## Methods

The CGH calculation for a 3D object comprised of PLSs is defined as:

1 $$\begin{aligned} u(x_h,y_h) = \sum _{j=1}^{N}\frac{A_j}{r_{hj}}\exp \left( i\frac{2\pi }{\lambda }r_{hj}\right) , \end{aligned}$$

where $u(x_h,y_h)$ is the complex-amplitude distribution on the hologram plane, which is a virtual plane used to simulate the wavefront from the PLSs. Here, *N* is the number of PLSs, *i* is the imaginary unit, $A_j$ is the amplitude of the *j*-th PLS, $\lambda$ is the wavelength of the object and reference beam, $r_{hj} =\{(x_h-x_j)^2+(y_h-y_j)^2+z_j^2\}^{1/2}$ is the distance between the *j*-th PLS and pixel $(x_h,y_h)$ of the hologram plane, and $(x_j,y_j,z_j)$ are the coordinates of the *j*-th PLS. In this system, a kinoform CGH^24^ is created from the complex-amplitude distribution on the hologram plane; i.e., CGH $c(x_h,y_h)$ is given by

2 $$\begin{aligned} c(x_h,y_h) = \left\lfloor \arg \{u(x_h,y_h)\}\frac{2^b}{2\pi } \right\rfloor , \end{aligned}$$

where $\arg (\cdot )$ is the operator for taking the argument of a complex number, $\lfloor \cdot \rfloor$ is the floor operator (rounding down), and *b* is the bit-depth of the CGH. Here, we set $b = 8$. Note that our system can also be applied to amplitude-type CGHs by changing equation (2).

Equation (1) can be processed efficiently using the N-LUT method^1^. Defining the wavefront created by one PLS as

3 $$\begin{aligned} T(x_h,y_h,x_j,y_j,z_j)\equiv \frac{1}{r_{hj}}\exp \left( i\frac{2\pi }{\lambda }r_{hj}\right) , \end{aligned}$$

Equation (1) can be rewritten as

4 $$\begin{aligned} u(x_h,y_h) = \sum _{j=1}^{N}A_jT(x_h,y_h,x_j,y_j,z_j). \end{aligned}$$

Because *T* has a shift-invariant property around $(x_j,y_j)$ on the hologram plane, the N-LUT method precomputes *T* with respect to the possible values of $z_j$ and substitutes it directly into the calculating equation (4) by superimposing it on the hologram plane. Considering aliasing on the SLM, the effective radius of *T* on *z* is

5 $$\begin{aligned} {R_z = z\frac{\lambda }{(4p^2-\lambda ^2)^{1/2}},} \end{aligned}$$

where *p* is the pixel-pitch of the SLM. If $(x_h,y_h)$ is outside this effective radius, the superimposition process is ignored. Thus,

6 $$\begin{aligned} T(x_h,y_h,x_j,y_j,z_j) = {\left\{ \begin{array}{ll} \text {Equation } (3) &{} d_j\le R_{z_j}, \\ 0 &{} otherwise, \end{array}\right. } \end{aligned}$$

where $$d_j=\{(x_h-x_j)^2 + (y_h-y_j)^2\}^{1/2}$$.

For a PLS with constant amplitude aligned on a straight line in a plane at a given *z*, the wavefronts on the hologram plane converge to a 1D wavefront. Given that the PLSs with $A_j=1$ are aligned from $-\infty$ to $+\infty$ on the *x*-axis, the wavefront on a line parallel to the *y*-axis at a given $x_h$ becomes

7 $$\begin{aligned} L(y_h,z) = \sum _{x_j=-R_{z_j}}^{R_{z_j}}T(x_h,y_h,x_j,0,z), \end{aligned}$$

where $-R_{z}\le y_h \le R_{z}$. According to equation (7), when the line length is longer than $2R_{z}$, the wavefront on a line parallel to the *y*-axis at $x_h$ that is further than $R_{z}$ from both end points is replaced by *L*. Note that *L* is invariant to rotations and horizontal movements of the line.

The CG-line method approximately replaces the wavefront with *L* even when the line length and position do not meet the conditions described above or the when the PLSs are aligned on a curve. In other words, the CGH computation is replaced by superimposing *L* in the normal direction at each point of the line. Note that in the other sections of this paper, we use the abbreviation 1DWF to represent *L*.

Figure 1(b) shows an overview of the CG-line method. If $f_z(x_h)$ is a function of a line-drawn object on *z* projected on the hologram plane, $\vec {n}_{z}(x_h)$ is a unit vector normal to the curve $f_z(x_h)$, ${\vec {h}}_{z}(x_h)\equiv [x_h,f(x_h)]$, and ${\vec {x}}_{h}=(x_h,y_h)$, the CGH calculation of equation (1) becomes

8 $$\begin{aligned} u(x_h,y_h) = \sum _{z}\sum _{x}L\{\vec {n}_{z}(x)\cdot {\vec {x}}_{h},z\}. \end{aligned}$$

Here,

9 $$\begin{aligned} L\{\vec {n}_{z}(x)\cdot {\vec {x}}_{h},z\} = {\left\{ \begin{array}{ll} \text {Equation }(7) &{} \vec {n}_{z}(x) \parallel \vec {d} \text { and } |\vec {n}_{z}(x)\cdot {\vec {x}}_{h}| \le R_z, \\ 0 &{} otherwise, \end{array}\right. } \end{aligned}$$

where $$\vec {d}={\vec {x}}_{h}-{\vec {h}}_{z}(x)$$.

In this system, the input coordinates from the drawing tablet are sampled at adjustable intervals as control points of the curve, and they are functionalized using the Catmull-Rom spline function, which is an interpolation polynomial that can generate smooth curves through the control points. Since the last two or three points can be used to define a curve, this method is ideal for our system, which displays the pen-input trajectory sequentially. The implementation is based on open-source software^26^. Furthermore, the computational burden is reduced by applying inter-frame subtraction to lines that are continuously inputted. In other words, the algorithm calculates only the complex-amplitude distributions corresponding to the lines added since the last frame update and accumulates them.

**Figure 10:**
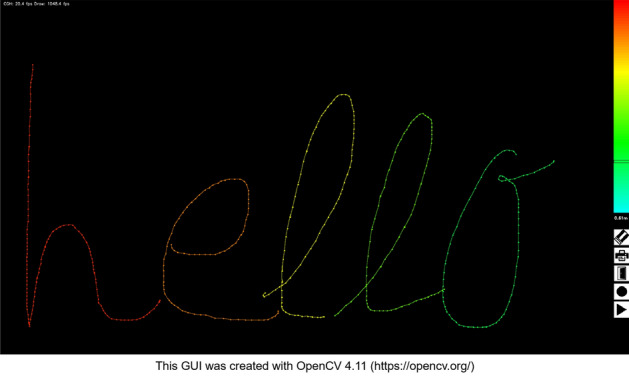
GUI display for this system.

The software used in this system has a Graphical User Interface (GUI) input built with OpenCV 4.11^27^. Figure 10 shows an example of the GUI display. The user selects the reconstruction depth of the 3D image from the color bar displayed on the right side of the drawing-tablet and draws the object to be displayed on the SLM. The color of the locus on the tablet corresponds to the depth. Based on the input-coordinate information, the software computes the curve function and superimposes the appropriate 1DWF in the normal direction at each point on the curve. These processes are parallelized with OpenMP, and the proposed system runs simultaneously on all cores of the CPU.

## Supplementary Information

Supplementary information 1.

Supplementary information 2.

## Data Availability

The datasets generated during and/or analyzed during the current study are available from the corresponding author on reasonable request.
